# Added value of positive intraluminal contrast CT over fluoroscopic examination for detecting gastrointestinal leakage after gastrointestinal surgery

**DOI:** 10.1038/s41598-024-51556-z

**Published:** 2024-01-10

**Authors:** Min Gwan Kim, Se Hyung Kim, Sun Kyung Jeon, Seungchul Han

**Affiliations:** 1 Department of Radiology, Seoul National University Hospital, 101 Daehakro, Jongro-Gu, Seoul, 03080 Korea; 2 Department of Radiology, Seoul National University College of Medicine, Seoul, Korea; 3 Institute of Radiation Medicine, Seoul National University Medical Research Center, Seoul, Korea; 4 Department of Radiology, Samsung Medical Center, Seoul, Korea

**Keywords:** Risk factors, Gastrointestinal system, Gastrointestinal diseases

## Abstract

We aimed to evaluate the added value of positive intraluminal contrast computed tomography (CT) over fluoroscopy in detecting anastomotic leakage after gastrointestinal (GI) surgery. A total of 141 GI surgery patients who underwent fluoroscopic examination and CT were included. Two radiologists reviewed the fluoroscopic images with and without CT to determine anastomotic leakage on a 5-point confidence scale and graded the leakage on a 4-point grading system. The hospital stay duration and treatment type were recorded. The radiologists’ diagnostic performance in determining leakage was compared using the receiver operating characteristics analysis, and interobserver agreement was analyzed. Fifty-three patients developed GI leakage. When CT was added to the fluoroscopic images, the area under the curve (AUC) values significantly increased for both reviewers. The interobserver agreement for leakage between the two reviewers was excellent and improved with the addition of CT (weighted kappa value, 0.869 versus 0.805). Postoperative intervention was more frequently performed (*P* < 0.001), and patients with leakage had a significantly longer mean postoperative hospital stay (45 days vs. 27 days) (*P* = 0.003). Thus, positive intraluminal contrast CT provides added value over fluoroscopic examination for detecting GI leakage in patients undergoing GI tract surgery, increasing AUC values, and improving interobserver agreement.

## Introduction

Leakage is a severe postoperative complication of gastrointestinal (GI) surgery. Early leakage identification is important in determining patient resuscitation because if the leak is not adequately recognized or delayed, it can cause organ contamination, leading to sepsis, multi-organ failure, and ultimately death^1–6^. The reported incidence of this complication varies between 1 and 29%, and mortality and morbidity range from 3%–39% and 29%–38%, respectively^1,7–9^.

Fluoroscopic examination with water-soluble intraluminal contrast agents is the modality of choice to confirm anastomotic patency and determine leakage before oral feeding can be initiated. Although fluoroscopic examinations using intraluminal contrast agents are highly specific, they are limited by their low sensitivity^7^. The fluoroscopic examination is sufficient to diagnose GI leakage in severe or obvious cases; however, in mild or indeterminate cases, it is difficult to accurately identify anastomotic leakage or the extent of leakage using fluoroscopy^10–12^. In such challenging cases, an additional non-intravenous (IV) contrast computed tomography (CT) with a water-soluble intraluminal contrast agent can be applied, as CT using a positive intraluminal contrast has several benefits, including being easy to perform in very ill patients and allowing for the determination of leakage through accumulated extraluminal contrast agent detection^13^. At our institute, fluoroscopic examination is the primary modality for diagnosing leakage after GI surgery, and positive intraluminal contrast CT is selectively used as a supplementary modality in indeterminate cases with fluoroscopic examination alone. To our knowledge, the added value of positive intraluminal contrast CT over fluoroscopic examinations has yet to be investigated.

We aimed to determine whether positive intraluminal contrast CT has any added value over fluoroscopic examination for detecting GI leakage in patients who have undergone GI surgery. We investigated whether other factors, such as the location of surgery and the grade of leakage, could influence the detection performance of positive intraluminal contrast CT.

## Materials and methods

This study was approved by the Institutional Review Board of Seoul National University Hospital (IRB no. 2007–060-1140). The requirement for informed consent was waived by the Institutional Review Board of Seoul National University Hospital because of the retrospective nature of the study. All the methods were performed in accordance with the relevant guidelines and regulations.

### Patient population

**Figure 1:**
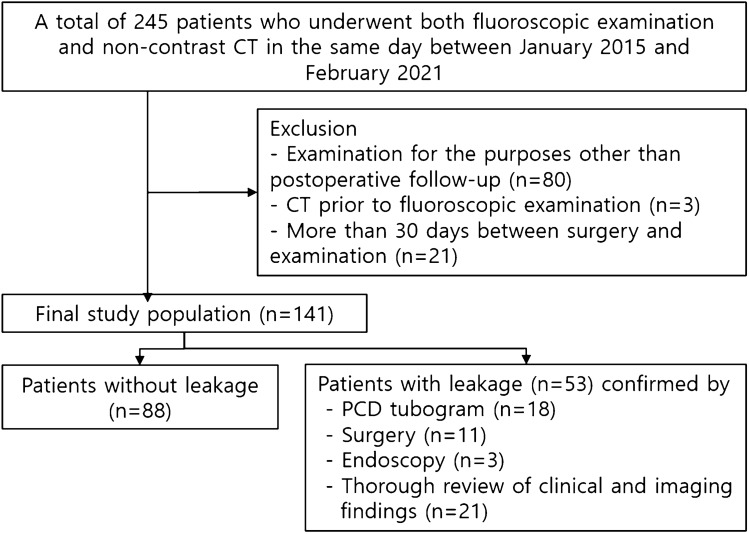
Flow chart showing patient enrollment.

From a radiological database, we identified 245 patients who underwent fluoroscopic examination and non-IV contrast CT on the same day between January 2015 and February 2021. After careful review of the electronic medical records (EMR), 104 patients were excluded for the following reasons: (1) Eighty patients underwent examinations for purposes other than postoperative follow-up, such as obstruction (due to ileus or tumor), perforation unrelated to surgery (due to endoscopic examination or tumor), or fistula (due to radiation therapy or remote surgery); (2) Three patients underwent CT before fluoroscopic examination; and 3) Twenty-one patients with more than 30 days between surgery and examinations. Figure 1 shows a flowchart of patient enrollment.

A total of 141 patients were included in this analysis. There were 94 men and 47 women with a mean age of 63 (28–91 years). The following clinical data were recorded by a radiologist with 3 years of experience: date of surgery, fluoroscopy and CT date, location and type of surgery, type of management for leakage (observation, percutaneous catheter drainage, endoscopic clipping, or reoperation), and discharge date.

### Imaging acquisition

The fluoroscopic examination was performed using SONIALVISION G4 (Shimadzu Corporation). The water-soluble intraluminal contrast agent used was gastrografin (Bayer AG). The volume of contrast agent used was 30–40 ml for the upper GI series, 100 ml for the small bowel follow-up examination, and 200–400 ml after 1:1 dilution for the colon study. Gastrografin was used as an undiluted solution for upper GI series and small bowel follow-through examinations.

After fluoroscopic examination, non-contrast CT is usually recommended and performed for the following patients: (1) patients in whom the result of fluoroscopic examination is equivocal; (2) patients in whom the site of leakage is not localized; (3) patients in whom there is strong clinical symptoms of leakage, such as persistent fever or leukocytosis, albeit with no definite leakage on fluoroscopic examination; and (4) patients in whom leakage is visible on fluoroscopy, but the leakage site is not clear.

If non-contrast CT was indicated, patients were transferred to the CT room immediately after fluoroscopic examination in the supine position. The mean time interval (± standard deviation [SD]) between fluoroscopic and CT examinations was 64.5 min (± 84.8 min): 48.9 ± 59.8 min for the stomach, 76.6 ± 96.5 min for the small bowel, and 73.4 ± 77.0 min for the colon. CT examinations were performed using several multidetector CT scanners. Details regarding the CT scanners and acquisition protocols are described in the Supplementary Material.

### Image analysis

Two abdominal radiologists (with 6 and 9 years of experience in abdominal imaging) blinded to the surgical and clinical results were recruited for the independent review sessions. In the first session, the radiologists independently reviewed the fluoroscopic images for the determination of GI leakage on a 5-point confidence scale: 1 = definitely absent, 2 = possibly absent, 3 = possibly present, 4 = probably present, and 5 = definitely present. In the second session, the patients were provided with positive intraluminal contrast CT images in addition to fluoroscopic images and the scores for GI leakage determination on a 5-point confidence scale. Two separate interpretation sessions were scheduled at 2-week intervals to minimize recall bias, and the images were presented randomly.

**Figure 2:**
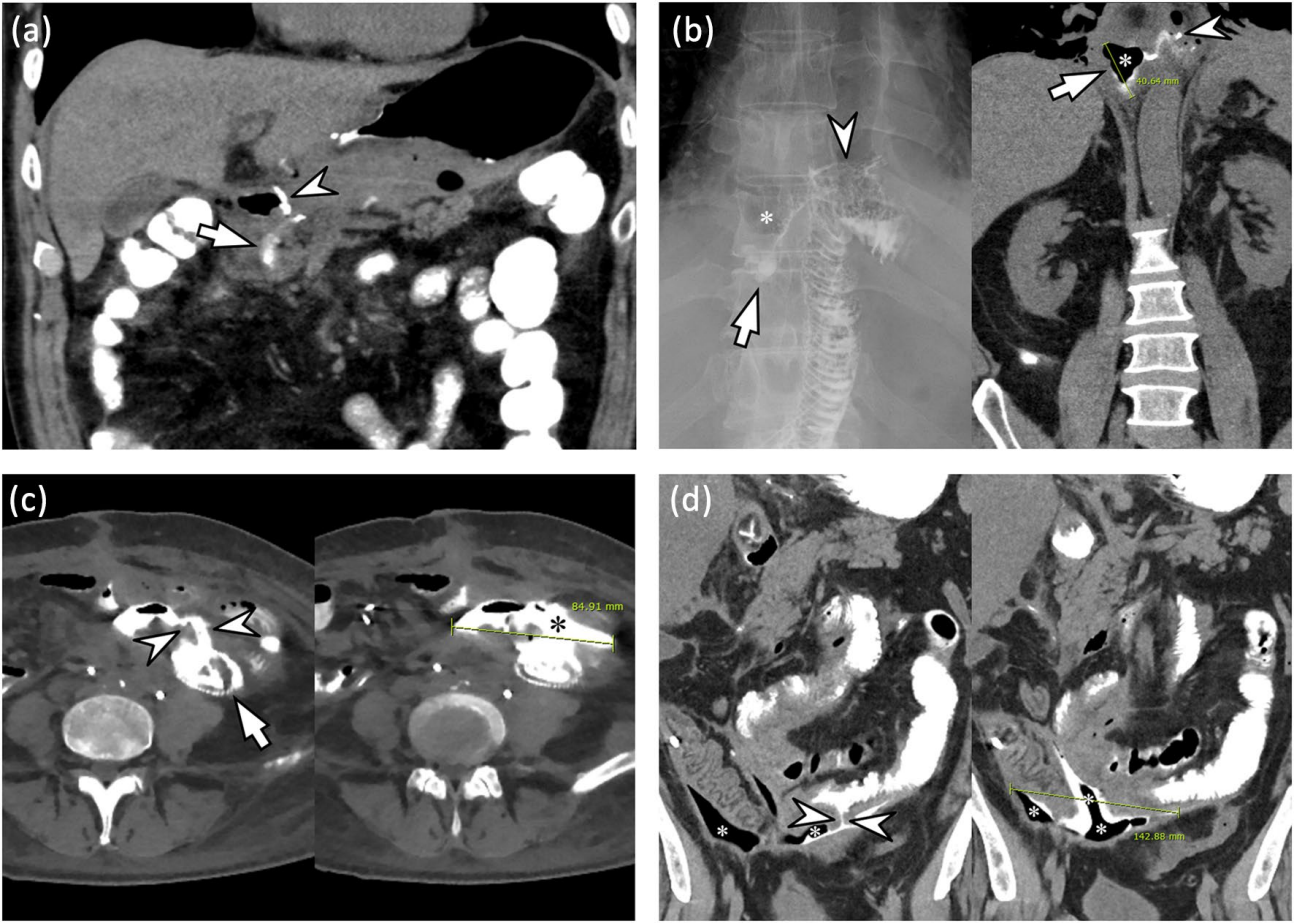
Representative examples for grading gastrointestinal leakage after gastrointestinal surgery. (**a**) Grade 1: minimal (pinpoint or scanty < 2 cm fluid). The patient underwent distal gastrectomy with Billroth I anastomosis for stomach cancer. On a coronal non-contrast CT image obtained immediately after fluoroscopic examination, there is a scanty (< 2 cm) extraluminal contrast collection (arrow) at the inferior aspect of gastroduodenostomy (arrowhead). (**b**) Grade 2: small (single and 2–5 cm fluid collection). The patient underwent total gastrectomy with esophagojejunostomy for stomach cancer. The fluoroscopic image (left) and coronal non-contrast CT image (right) obtained after fluoroscopic examination reveals contrast leakage (arrow) at the right side of the esophagojejunostomy site (arrowhead). Note a 4 cm small air-containing cavity (*) with small contrast material around the leakage site. (**c**) Grade 3: intermediate (5–10 cm fluid collection). The patient underwent metallic stent insertion for sigmoid colon obstruction due to sclerosing mesenteritis. On axial non-contrast CT images obtained immediately after fluoroscopic examination, there is a contrast leakage (arrowheads) from proximal end of metallic stent (arrow) at sigmoid colon. Note an 8.5 cm fluid cavity (*) filled with extravasated contrast material around the leakage site. (**d**) Grade 4: large (> 10 cm or disseminated fluid collection). The patient underwent right hemicolectomy for ascending colon cancer. On coronal non-contrast CT images obtained immediately after fluoroscopic examination, there is a small defect at ileum (arrowheads). Note a > 14 cm large air and fluid filled cavity containing extravasated contrast material (*) around the leakage site.

In both review sessions, for patients who had suspicious leakage on images, the reviewers were asked to localize the leakage site and grade the leakage using a 4-point grading system according to the amount of extraluminal fluid collected on non-IV contrast CT images: 1, minimal (pinpoint or scanty < 2 cm fluid); 2, small (single and 2–5 cm fluid collection); 3, intermediate (5–10 cm fluid collection); and 4, large (> 10 cm or disseminated fluid collection). To accurately measure the size of contrast leakage, all three CT plane images were carefully reviewed. After an independent review, discrepancies regarding the presence and grade of GI leakage between the two reviewers were resolved through consensus between the two reviewers and another senior reviewer (with 21 years of experience in abdominal imaging). Representative images of each leakage grade are shown in Fig. 2. The reviewers further recorded the location of the leakage.

### Diagnosis of leakage

Two other radiologists (with 14 and 21 years of experience in abdominal imaging) carefully reviewed the fluoroscopic images, positive intraluminal contrast CT, patients’ EMR, and picture-achieving communication systems to confirm the presence, location, and grade of GI leakage. The presence or absence of GI leakage was confirmed through percutaneous catheter drainage (PCD) tubography, surgery, endoscopic examination, or an integrative approach with fluoroscopic imaging and CT findings. The leakage location was divided into three groups: stomach, small bowel, and colon.

### Statistical analysis

Among patients with and without GI leakage, continuous variables were compared using the independent *t*-test, and categorical variables were compared using the chi-square or Fisher’s exact tests. The rates of leakage management and mortality were compared using the chi-square test. Interobserver agreement for the presence and grade of leakage was assessed using weighted kappa statistics. Details of the statistical analyses are provided in the Supplementary Material.

The individual diagnostic performances of the two independent reviewers for leakage detection were evaluated using receiver operating characteristics (ROC) curve analysis. A pairwise comparison of the area under the curve (AUC) values was performed between two separate review sessions using DeLong's method to assess improvements in radiologists’ performance with positive intraluminal contrast CT. Each reviewer performed a subgroup analysis of the ROC curve for the three groups (stomach, small bowel, and colon).

For the leakage grades determined by consensus, the rates of postoperative interventional management and hospital stay duration after surgery were compared using chi-square and ANOVA tests, respectively.

All statistical analyses were performed using SPSS version 25.0 (IBM Corp.) and MedCalc version 11.6. software (MedCalc Software). Statistical significance was set at *P* < 0.05.

## Results

Of the 141 patients, 53 (37.6%) were confirmed to have GI leakage (Table 1), of whom 18 were confirmed to have leakage through PCD tubography, 11 through surgery, and 3 through endoscopy. Leakage was confirmed in the remaining 21 patients through a thorough review of clinical, laboratory, and imaging findings. The remaining 88 patients were diagnosed with no leaks. Of these, 14 patients were confirmed to have no leakage through PCD tubography, and 74 patients were confirmed to have no leakage through a thorough review of clinical, laboratory, and imaging findings.

**Table 1:** Demographics and clinical characteristics of all patients.

		Leakage (n = 53)	No leakage (n = 88)	*P* value
Age (mean ± SD, years)		59.3 ± 15.2	64.6 ± 14.3	**0.040**
Sex	Male	34 (64.2)	60 (68.2)	0.623
	Female	19 (35.8)	28 (31.8)	
Location of surgical site	Stomach	22 (41.5)	38 (43.2)	0.322
	Small bowel	27 (50.9)	48 (54.5)	
	Colon	4 (7.5)	2 (2.3)	
Postoperative intervention	Percutaneous drainage	30 (56.6)	14 (15.9)	** < 0.001**
	Endoscopic clipping	1 (1.9)	0 (0)	
	Reoperation	11 (20.8)	0 (0)	
Mean period ± SD between (days)	Surgery and examination	13.3 ± 7.9	9.5 ± 6.5	** < 0.001**
	Surgery and discharge	44.6 ± 22.1	27.2 ± 21.0	**0.003**
	Examination and discharge	31.4 ± 21.4	17.7 ± 19.4	** < 0.001**
Mortality	7 (13.2)	2 (2.7)	**0.027**	

Data are the number of patients unless specified. Data in parentheses indicate percentage.

*P* values written in bold indicate a statistical significance.

Table 1 presents the statistical results for the demographics and clinical findings of patients with and without GI leakage. The mean age ± SD was significantly lower in patients with leakage (59.3 ± 15.2 years) than in those without leakage (64.6 ± 14.3 years) (*P* = 0.040). The rate and type of management were significantly different between patients with and without GI leakage; postoperative intervention was performed more frequently in patients with leakage (79.2%, 42/53) than in those without leakage (15.9%, 14/88) (*P* < 0.001). The type of management was significantly different between the two groups (*P* < 0.001). The mean duration ± SD of postoperative hospital stay was significantly longer in patients with leakage (44.6 ± 22.1 days) than in those without (27.2 ± 21.0 days) (*P* = 0.003). The mortality rate was also significantly higher in patients with leakage (13.2%, 7/53) than in those without leakage (2.7%, 2/88) (*P* = 0.027).

Table 2 summarizes the confidence scores and GI leakage grades reported by the two reviewers. The weighted *k*-value (0.869) for leakage between the two reviewers was excellent and increased when positive intraluminal contrast CT was added to the fluoroscopic images (weighted *k*-value = 0.805). Regarding the leakage grade, the weighted *k*-value (0.556) between the two reviewers also increased when positive intraluminal contrast CT was added to the fluoroscopic images (weighted *k*-value = 0.426).

**Table 2:** Results of confidence score and grade for gastrointestinal leakage by two reviewers.

Confidence score for the presence of leakage*													
Fluoroscopy only		Reviewer 1					Fluoroscopy + CT		Reviewer 1				
		1	2	3	4	5			1	2	3	4	5
Reviewer 2	1	0	0	0	0	0	Reviewer 2	1	63	0	0	1	1
	2	86	9	1	7	1		2	28	2	0	1	3
	3	4	0	0	4	2		3	0	0	0	1	2
	4	3	1	0	5	0		4	0	0	0	2	2
	5	0	1	0	2	15		5	1	0	0	1	33
Grade of gastrointestinal leakage^†^													
Fluoroscopy only		Reviewer 1					Fluoroscopy + CT		Reviewer 1				
		1	2	3	4				1	2	3	4	
Reviewer 2	1	5	4	0	0		Reviewer 2	1	7	7	0	0	
	2	1	4	3	0			2	3	7	3	2	
	3	2	3	4	1			3	1	2	4	1	
	4	0	1	0	0			4	0	1	0	2	

*For the presence of gastrointestinal leakage, two reviewers independently scored images on a 5-point confidence scale: 1, definitely absent; 2, possibly absent; 3, possibly present; 4, probably present; 5, definitely present. † Reviewers graded the leakage on a 4-point grading system: 1, minimal (focal, pin-point); 2, mild (< 5 mm); 3, moderate (5–15 mm); 4, severe (> 15 mm).

**Figure 3:**
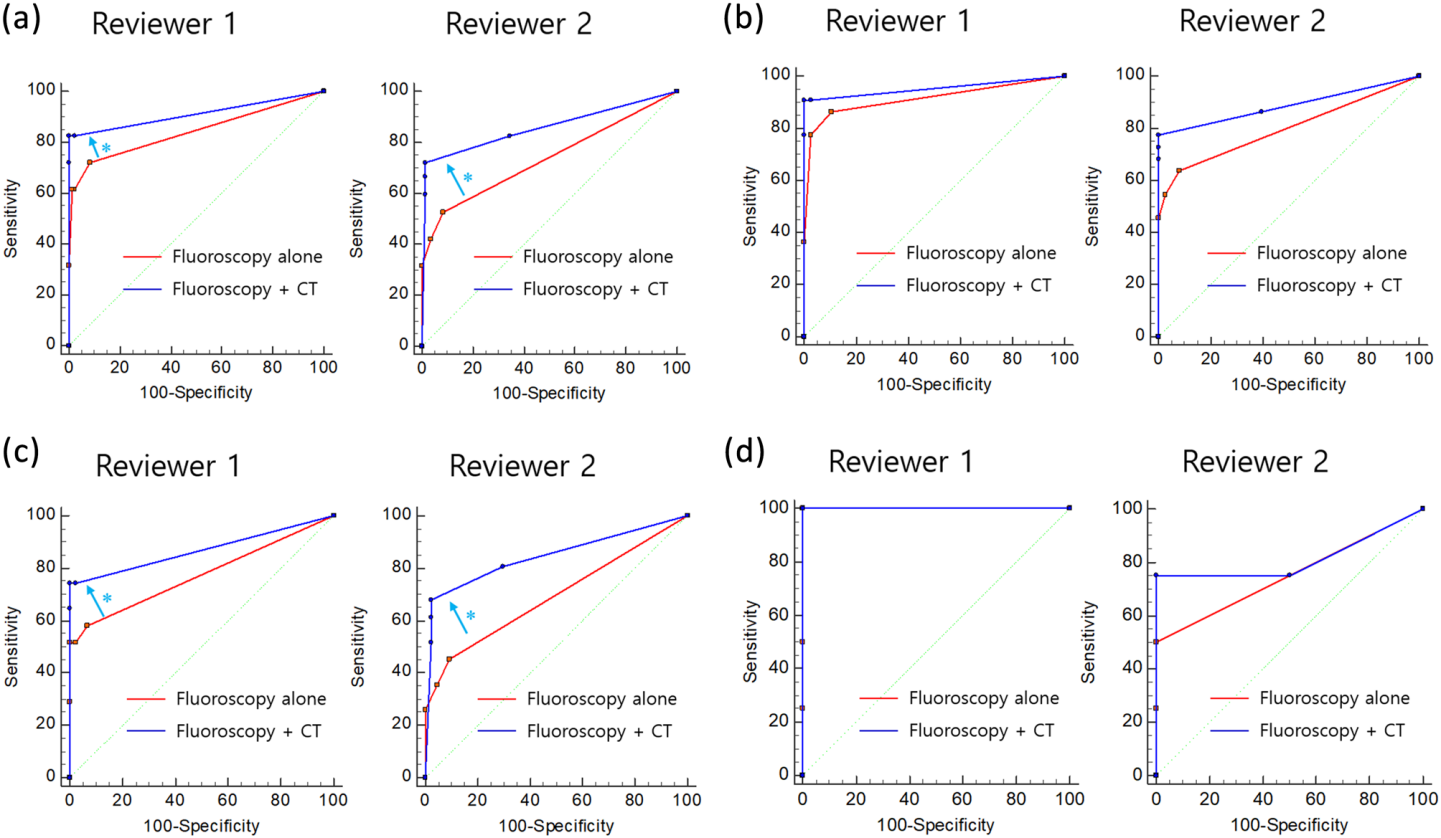
Results of the ROC analysis to detect gastrointestinal leakage. (**a**) For all 141 patients, when positive enteric contrast CT images were added to fluoroscopic images, the AUC values increased from 0.859 to 0.942 for reviewer 1 and from 0.757 to 0.879 for reviewer 2, and the difference was statistically significant in both reviewers (*P* = 0.001 for reviewer 1 and *P* = 0.002 for reviewer 2). (**b**–**d**) In a subgroup analysis according to the location of surgery, AUC values for the three groups (stomach [**b**], small bowel [**C**], and colon [**d**]) were increased when positive enteric contrast CT images were added to fluoroscopic images. However, the difference was statistically significant only in the small bowel group (**c**) (from 0.763 to 0.856 for reviewer 1, *P* = 0.026; from 0.684 to 0.828 for reviewer 2, *P* = 0.011). Asterisks (*) indicate a statistically significant increase in AUC values.

**Figure 4:**
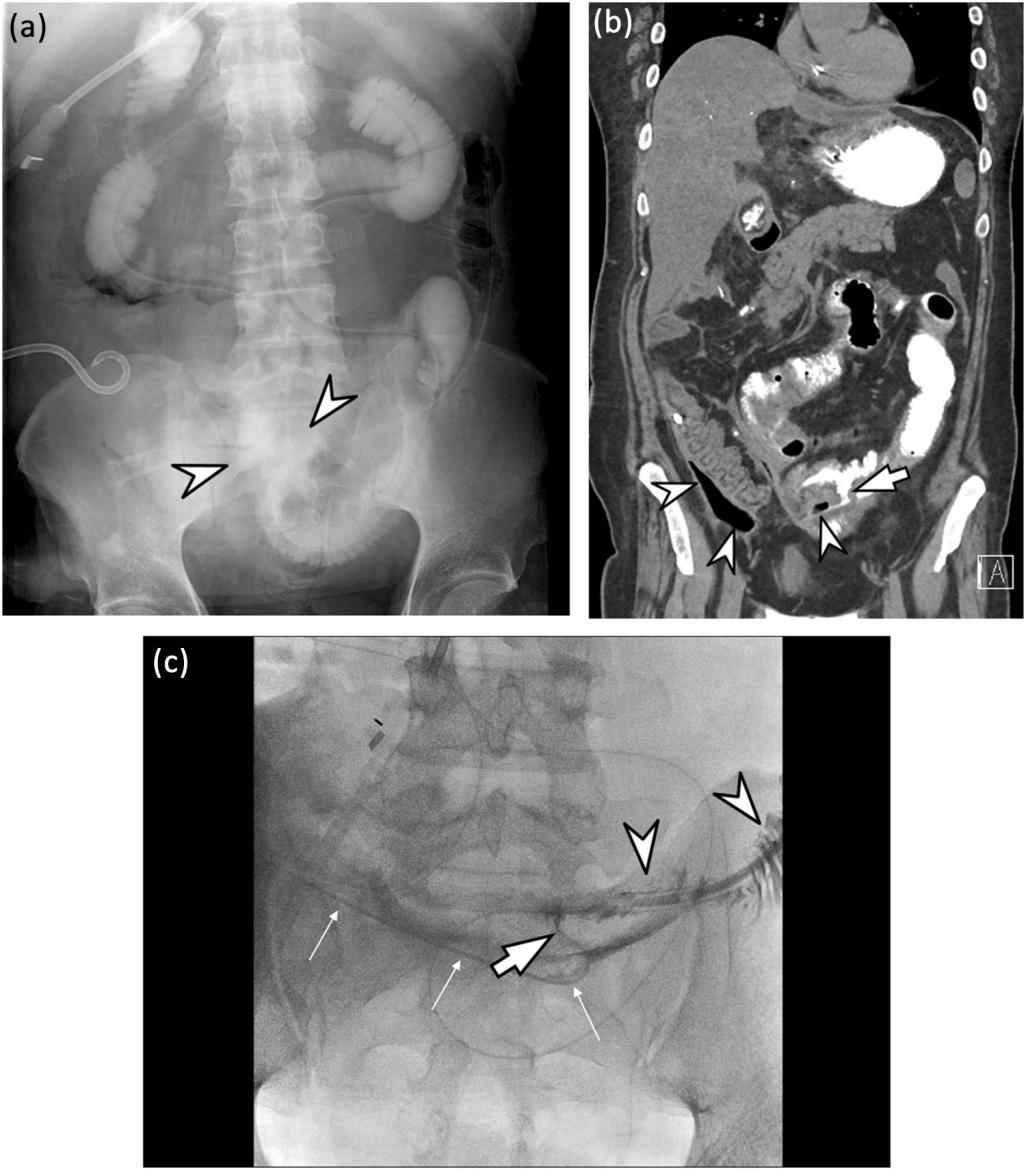
A 40-year old man who underwent primary repair operation for small bowel perforation. After operation, the patient complained of abdominal pain and persistent fever. (**a**) On small bowel follow-through examination after taking a 100 ml of oral iodinated contrast agent (gastrografin®), a fuzzy-marginated, increased attenuating area (arrowheads) in the pelvic cavity is observed. However, two radiologists were not able to determine the presence of leakage and to localize the leakage site. Therefore, they scored 3 (possibly present) for the presence of leakage. (**b**) On a coronal non-contrast CT image obtained immediately after fluoroscopic examination, a small & mild defect (arrows) is clearly demonstrated at the inferior aspect of ileum. There is also a moderate amount of air-containing cavity with extravasated contrast material (arrowheads) around the leakage site. Both radiologists scored 5 (definitely present) for the presence of leakage. (**c**) On a tubogram obtained after contrast injection through a percutaneous catheter drainage (PCD) tube (thin arrows), a small defect of the ileal wall (arrow) is well demonstrated and the ileal lumen is filled with inserted contrast materials (arrowheads).

**Figure 5:**
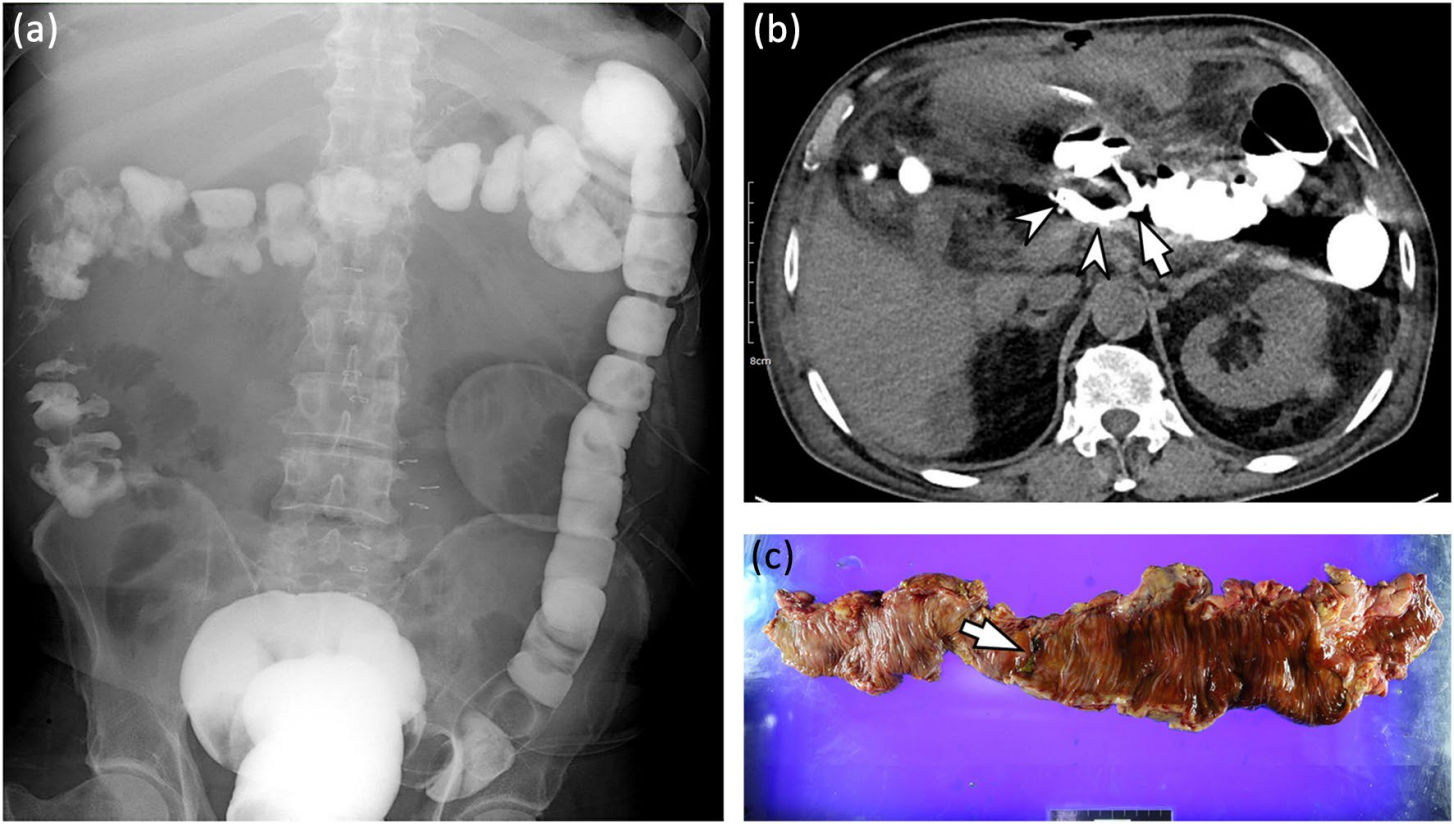
A 64-year old man who underwent distal pancreatectomy for a pancreatic neuroendocrine tumor. During operation, there was an adhesion around the transverse colon. During adhesiolysis, a colonic tear occurred and transverse colon segmental resection and anastomosis were performed. After the operation, the patient complained of sepsis. (**a**) On a single contrast colon study image obtained after inserting 200 ml of a 1:1 diluted enteric iodinated contrast agent (gastrografin®), there is no evidence of leakage in the entire colon. Therefore, two radiologists scored 1 (definitely absent) for the presence of leakage. (**b**) On an axial non-contrast CT image obtained immediately after fluoroscopic examination, a small defect (arrow) at posterior aspect of colonic anastomotic site can be observed. Note the small amount of extravasated contrast material (arrowhead) around operative site. Both radiologists scored 5 (definitely present) for the presence of leakage. The patient underwent re-operation. On the operative field, there is a defect at colonic anastomotic site and subtotal colectomy was finally performed. (**c**) On a photograph of gross specimen, there is an ovoid perforation site (arrow) at the transverse colon.

**Figure 6:**
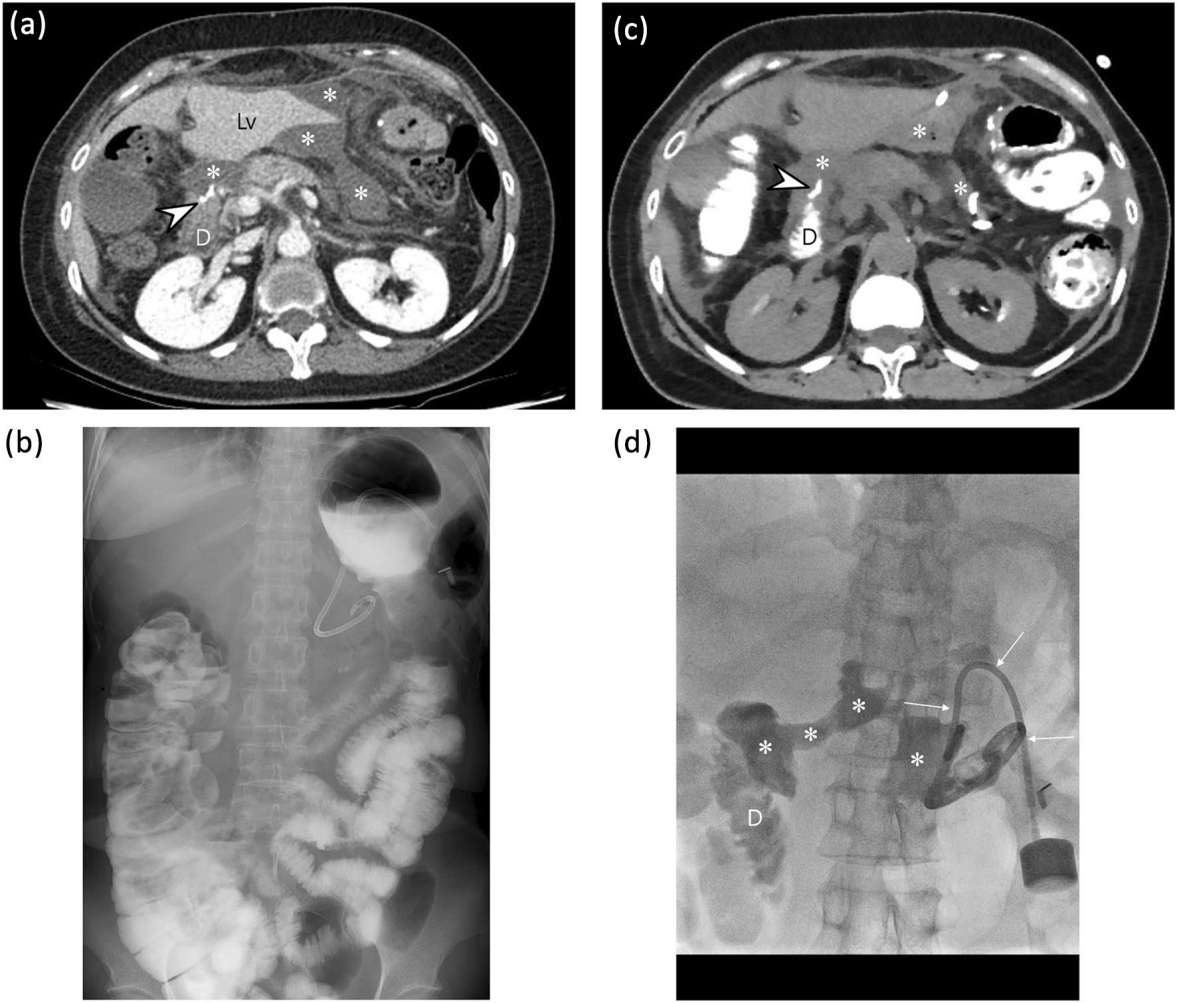
A 49-year old woman who underwent distal gastrectomy with Billroth II anastomosis for early gastric cancer. Ten days after the operation, the patient visited an emergency department due to severe abdominal pain. (**a**) On portal phase contrast-enhanced CT, multiple fluid collection (*) around the duodenal stump (D) and the liver (Lv) is observed. Therefore, leakage at the duodenal stump was highly suspected. (**b**) On a delayed fluoroscopic image obtained during upper gastrointestinal series using 200 ml of a 1:1 diluted enteric iodinated contrast agent (gastrografin®), there is no definite leakage. Two radiologists scored this patient as 1 (definitely absent) for the presence of leakage. (**c**) On an axial non-contrast CT image obtained immediately after fluoroscopic examination, there is no definite contrast extravasation around the duodenal stump (D) or within several fluid collections (*). Therefore, both radiologists scored this patient as 1 (definitely absent) for the presence of leakage. (**d**) On a tubogram obtained after contrast injection through a percutaneous catheter drainage (PCD) tube (thin arrows), the duodenal 2nd portion (**d**) is opacified, along with multiple fluid collection (*). Therefore, duodenal stump leakage was finally diagnosed. This is a case of false-negative leakage on both fluoroscopy and positive enteric contrast CT.

Table 3 and Fig. 3 list the individual performances of the two radiologists in detecting leakages during two successive independent review sessions. When positive intraluminal contrast CT images were added to the fluoroscopic images, the AUC for all 141 patients significantly increased from 0.859 to 0.942 for reviewer 1(*P* = 0.001) and from 0.757 to 0.879 for reviewer 2(*P* = 0.002). In the subgroup analysis according to surgery location, the AUC values for the three groups (stomach, small bowel, and colon) increased when positive intraluminal contrast CT images were added to the fluoroscopic images. The difference was statistically significant only in the small bowel group (from 0.763 to 0.856 for reviewer 1, *P* = 0.026; from 0.684 to 0.828 for reviewer 2, *P* = 0.011). Representative examples are shown in Figs. 4, 5, 6.

**Table 3:** ROC Results for Detecting Gastrointestinal Leakage of Fluoroscopy Without and With Positive Intraluminal Contrast CT.

			Fluoroscopy only	Fluoroscopy + CT	*P* values
For all patients (n = 141)	AUC values	Reviewer 1	0.859	0.942	**0.001**
		Reviewer 2	0.757	0.879	**0.002**
	Accuracy (%)	Reviewer 1	85.8 (121/141)	95.74 (135/141)	**0.004**
		Reviewer 2	78.7 (111/141)	90.78 (128/141)	**0.005**
Stomach group (n = 60)	AUC values	Reviewer 1	0.913	0.953	0.138
		Reviewer 2	0.798	0.887	0.067
	Accuracy (%)	Reviewer 1	90.0 (54/60)	96.7 (58/60)	0.145
		Reviewer 2	81.7 (49/60)	91.7 (55/60)	0.109
Small bowel group (n = 75)	AUC values	Reviewer 1	0.763	0.856	**0.026**
		Reviewer 2	0.684	0.828	**0.011**
	Accuracy (%)	Reviewer 1	84.0 (63/75)	94.7 (71/75)	**0.035**
		Reviewer 2	77.3 (58/75)	90.7 (68/75)	**0.026**
Colon group (n = 6)	AUC values	Reviewer 1	1.000	1.000	1.000
		Reviewer 2	0.750	0.813	0.712
	Accuracy (%)	Reviewer 1	66.7 (4/6)	100.0 (6/6)	0.138
		Reviewer 2	66.7 (4/6)	83.3 (5/6)	0.523

*ROC* receiver operating characteristic, *AUC* area under the curve.

At the first session, only fluoroscopic images were provided for the detection of gastrointestinal leakage. At the second session, however, two reviewers were provided positive enteric contrast CT in addition to fluoroscopic images. Numbers in parenthesis are proportion of patients.

*P* values written in bold indicate a statistical significance.

After a consensus review of fluoroscopic and CT images between the two reviewers, 9 patients were designated as having grade 1 leakage, 11 with grade 2, 13 with grade 3, and 20 with grade 4 leakage. Although the rate of postoperative interventional management tended to be higher in patients with a higher grade of leakage, it was not significantly different among the grades of leakage, with rates of 66.7% (6/9) for grade 1, 81.8% (9/11) for grade 2, 76.9% (10/13) for grade 3, and 85% (17/20) for grade 4 (*P* = 0.716). In addition, there was no significant difference in the rate of postoperative interventional management between patients with grade 1 leakage (6/9, 66.7%) and those with grade 2–4 leakage (36/44, 81.8%) (*P* = 0.372). The mean duration ± SD of hospital stay after surgery was the longest in grade 4 (50.4 ± 22.8 days), followed by grade 2 (48.8 ± 21.8 days), grade 3 (76.9 ± 43.5 days), and grade 1 (66.7 ± 30.4 days), albeit with no statistical significance (*P* = 0.156). However, the mean duration ± SD of hospital stay in patients with grade 2–4 leakage was 47.6 ± 23.1 days, which was significantly longer than that in patients with grade 1 leakage (30.4 ± 6.2 days) (*P* = 0.033).

## Discussion

We found that non-IV contrast CT performed immediately after fluoroscopic examination has added value over fluoroscopic examination alone in detecting leakage after GI tract surgery. According to both reviewers, the AUC values for diagnosing GI leakage in all 141 patients significantly increased when CT images were added to the fluoroscopic images. This result is consistent with those of previous studies^1,7^. According to Kauv et al., inserting an intraluminal positive contrast agent for CT with a retrograde contrast enema allows for the accurate diagnosis of colorectal anastomotic leakage^1^. They also found that contrast extravasation on CT was the most specific sign for diagnosing anastomotic leakage^1^. IV contrast-enhanced CT without intraluminal contrast can provide valuable information regarding postoperative leakage after GI tract surgery. Indeed, free intra-abdominal fluid and perianastomotic stranding on IV contrast-enhanced CT are sensitive indicators of anastomotic leakage^7^. Samji et al. reported that the leakage of intraluminal contrast agents on CT is a precise imaging predictor (96.6%), and the diagnostic performance for detecting anastomotic leakage is high when intraluminal contrast agents are used during CT^7^. Considering that using an IV contrast agent during CT can trigger serious side effects such as anaphylaxis, non-IV contrast CT using an intraluminal contrast agent may be noteworthy.

In the subgroup analysis, we observed that the AUC value for diagnosing anastomotic leakage significantly increased with CT only in a subset of patients who underwent small bowel surgery. This result is in good agreement with our expectations. The exact identification of anastomotic leakage after GI tract surgery may be hindered by overlapping bowel loops on fluoroscopic examination. In contrast, CT, a cross-sectional imaging technique, allows direct visualization of both intraluminal and extraluminal structures^14^. Such difficulty could be maximized on fluoroscopic examination when evaluating the small bowel compared with the stomach and colon, which are usually not eclipsed by the surrounding bowel loops, even under fluoroscopic examination. If fluoroscopic findings are equivocal and inconclusive in patients who have undergone small bowel surgery and have suspected anastomotic leakage based on clinical and laboratory findings, acquisition of non-IV contrast CT is strongly recommended after fluoroscopic examination.

We also found that postoperative interventional procedures were performed more frequently in patients with anastomotic leakage than in those without. The mean duration of the postoperative hospital stay (45 days) was significantly longer in patients with leakage than in those without leakage (27 days) (*P* = 0.003). Considering the higher event-related mortality rate in patients with leakage (13.2%, 7/53) than in those without leakage (2%, 2/88), accurate identification and subsequent prompt intervention for anastomotic leakage are of great clinical importance.

We also observed a significant difference in the duration of hospital stay between patients with grade 1 leakage and those with grade 2–4 leakage. More specifically, the mean duration of hospital stay in patients with grade 2–4 leakage was 47.6 days, which was significantly longer than in patients with grade 1 leakage (30.4 days) (*P* = 0.033). However, there was no significant difference in the rate of postoperative interventional management between patients with grade 1 leakage (6/9, 66.7%) and those with grade 2–4 leakage (36/44, 81.8%) (*P* = 0.372). Nonetheless, we observed a tendency toward an increased rate of postoperative interventional management in patients with a higher grade of GI leakage: 66.7% (6/9) for grade 1, 81.8% (9/11) for grade 2, 76.9% (10/13) for grade 3, and 85% (17/20) for grade 4; however, the difference was not statistically significant (*P* = 0.716). We believe that the small number of patients with GI leakage (n = 53) may be responsible for the weak statistical power and insignificant results regarding the rate of postoperative interventional management. Therefore, further studies enrolling more patients with GI leakage are warranted to test our hypothesis.

CT has limitations in identifying leakage^3^. If any leakage is located in a non-dependent portion, CT can miss it. Duodenal stump leakage after subtotal gastrectomy with Billroth II reconstruction or total gastrectomy with Roux-en-Y reconstruction may be another challenge in positive intraluminal contrast CT in evaluating leakage because the ingested contrast agent does not reach the stump level. In our study, these limitations should not play a major role in limiting CT because frequent position changes during previous fluoroscopic examinations and inevitable ambulation from the fluoroscopic room to the CT room may facilitate an even distribution of intraluminal contrast to the entire bowel loop, even in the duodenal stump. In our study, one patient underwent subtotal gastrectomy with Billroth II anastomosis and had duodenal stump leakage (Fig. 6). Leakage was correctly diagnosed with subsequent non-contrast CT but was missed on fluoroscopy. We believe that combining fluoroscopic examination and successive non-IV contrast CT may improve the imaging accuracy for diagnosing leakage after GI tract surgery.

We acknowledge the limitations of this study. First, there is a lack of firm reference standards for confirming leakages. In other words, the presence of leakage was not confirmed by direct visualization of the leakage during surgery, endoscopy, or tubography in all patients. However, we believe that this limitation is unavoidable because definite treatment procedures, such as PCD, endoscopic clipping, or surgery, are unnecessary when there is no evidence of leakage on imaging and no clinical signs, such as persistent fever and leukocytosis. Physicians use clinical findings and imaging features to exclude GI tract leakage. Leakage was confirmed in 21 patients through a thorough review of clinical, laboratory, and imaging findings. Furthermore, imaging studies may be a potential source of false-negative and false-positive diagnoses of GI leakage. Second, unlike a previous study in which the amount of extraluminal fluid was used to grade leakage^3^, we used the size of the leakage point on both fluoroscopy and CT. The use of leakage size for grading leakage may underestimate the severity of leakage, especially when the extraluminal fluid has a direct compressive effect on the leakage site. Combined interpretation using both the size of the leakage and the amount of extraluminal fluid should be performed to grade the leakage in future investigations. Third, radiological interpretation after fluoroscopic examination and CT may prompt surgeons to perform interventional treatment, increasing the rate of interventional therapy in patients with leakage on imaging. At our institution, postoperative interventions, including reoperation or endoscopic clipping, are usually planned after a thorough examination of the patient's clinical status and laboratory results. Hence, PCD was performed in 14 patients without leakage, and postoperative intervention was not performed in 11 patients even with leakage on imaging.

In conclusion, positive intraluminal contrast CT has added value to fluoroscopic examination for GI leakage detection in patients who have undergone GI surgery and have equivocal and discrepant results between clinical findings and fluoroscopic examination. The added value can be maximized for patients who have undergone small bowel surgery by significantly increasing the AUC values and improving the interobserver agreement.

### Supplementary Information

Supplementary Information.

## Data Availability

The datasets generated or analyzed during the study are available from the corresponding author on reasonable request.

## Abbreviations

GI: Gastrointestinal
IV: Intravenous
PCD: Percutaneous catheter drainage
SD: Standard deviation
EMR: Electronic medical records
