# Supplementary material for: Added value of positive intraluminal contrast CT over fluoroscopic examination for detecting gastrointestinal leakage after gastrointestinal surgery
Source: Sci Rep. 2024 Jan 10;14:1011. doi: 10.1038/s41598-024-51556-z (PMC10781980; doi:10.1038/s41598-024-51556-z)
Supplement: Supplementary file 1 — Supplementary Information. [file 41598_2024_51556_MOESM1_ESM.docx]

**Supplementary Materials**

**Materials and Methods**

*Imaging Acquisition*

CT examinations were performed using one of the following multi-detector CT scanners: Brilliance 64, Philips Medical Systems (n=25); IQon Spectral CT, Philips Medical Systems (n=43); Brilliance iCT 256, Philips Medical Systems (n=2); SOMATOM Force, Siemens Medical Systems (n=30); SOMATOM Definition, Siemens Medical Systems (n=38); Sensation 16, Siemens Medical Systems (n=2); and Aquilion ONE, Canon Medical Systems (n=1). Non-IV contrast CT images were obtained immediately after fluoroscopic examination. The following CT acquisition parameters were used: 100–120 kVp, 150–300 mAs, 2.8–11.5 mm detector collimation, 0.6–1.0 pitch, 0.33–0.75 second gantry rotation time, 3 mm slice thickness, and 3 mm reconstruction interval.

*Statistical Analysis*

Among patients with and without GI leakage, continuous variables, such as the age and duration of hospital stay (from the date of surgery to the date of discharge) were compared using the independent t-test, while categorical variables including the type (PCD, endoscopic clipping, and re-operation) of management for GI leakage were compared using the chi-square or Fisher exact tests. The rate of management for leakage and mortality rates were compared using the Chi-square test.

The interobserver agreement for the presence and grade of leakage was assessed using weighted kappa statistics. A k-value of 0 to 0.20 indicated poor agreement; 0.21 to 0.40, fair agreement; 0.41 to 0.60, moderate agreement; 0.61 to 0.80, good agreement; and 0.81 to 1.00, excellent agreement [14]. The kappa value (κ) for leakage grade was calculated for cases where leakage was detected by both reviewers, except for the case where one side of modalities was missed.
